# Power Output and Efficiency During Supine, Recumbent, and Upright Cycle Ergometry

**DOI:** 10.3389/fspor.2021.667564

**Published:** 2021-06-10

**Authors:** Anja Wehrle, Sarah Waibel, Albert Gollhofer, Kai Roecker

**Affiliations:** ^1^Institute for Exercise and Occupational Medicine, Faculty of Medicine, Medical Center, University of Freiburg, Freiburg, Germany; ^2^Institute of Sport and Sport Science, University of Freiburg, Freiburg, Germany; ^3^Department of Neurology and Neuroscience, Faculty of Medicine, Medical Center, University of Freiburg, Freiburg, Germany; ^4^Institute for Applied Health Promotion and Exercise Medicine (IfAG), Furtwangen University, Furtwangen, Germany

**Keywords:** CPET, cycling, posture, performance, ventilator efficiency, gross efficiency

## Abstract

Recumbent and supine cycling are common exercise modes in rehabilitation and clinical settings but the influence of postures on work efficiency is unclear. Therefore, the aim of this study was to compare metabolic and ventilatory efficiency during upright, recumbent, and supine postures. Potential differences should be assessed for suitable diagnostics and for prescriptions of training that probably is performed in alternative postures. Eighteen healthy subjects (age: 47.2 ± 18.4 years; 10 female, 8 male) participated in the study and each completed three incremental cycle ergometer tests until exhaustion in upright, recumbent (40°), and supine positions. Gas exchange, heart rate (HR), and lactate concentrations were analyzed and efficiency was calculated subsequently. Testing sessions were performed in random order within a 2-week period. Upright cycling resulted in significantly higher peak values [power output, oxygen uptake (Vo_2_), HR] as well as performance at lactate and ventilatory thresholds in comparison to recumbent or supine positions. Vco_2_/Vo_2_ slope and ventilatory efficiency (VE/Vco_2_ slope) were not affected by posture. Aerobic work efficiency (Vo_2_/P slope) and gross efficiency (GE) differed significantly between postures. Hereby, GE was lowest in supine cycling, particularly obvious in a mainly aerobic condition at 70 Watt [Median 11.6 (IQR 10.9–13.3) vs. recumbent: 15.9 (IQR 15.6–18.3) and upright: 17.4 (IQR 15.1–18.3)]. Peak power as well as GE and work efficiency values are influenced by cycling position, reinforcing the importance of adjusting test results for training prescriptions. Surprisingly, ventilatory efficiency was not affected in this study and therefore does not seem to falsify test results for pulmonary diagnostics.

## Introduction

Cycling is one of the most common method to assess and promote cardiorespiratory fitness in recreational sports, as well as in rehabilitation and clinical practice (Garber et al., 2011; American College of Sports Medicine et al., 2018). Cardiopulmonary exercise tests (CPET) play a major role in assessing physical capacity and obtaining useful clinical diagnosis and prognostic information (Guazzi et al., 2012, 2016; Arena et al., 2020) in patients with cardiovascular and pulmonary diseases. Cardiopulmonary exercise test results are used to prescribe adequate training intensities for appropriate training stimuli (Pedersen and Saltin, 2015) or evaluating the effectiveness of exercise interventions, especially in research settings.

**Figure 1:**
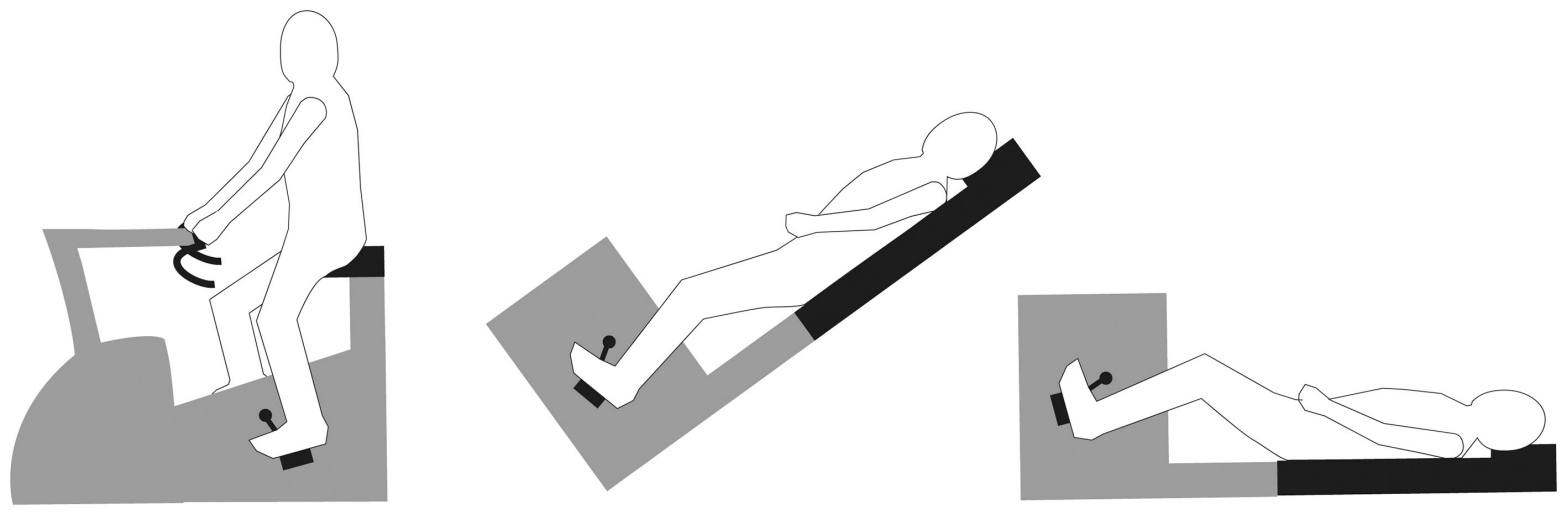
Illustration of the three different cycling postures.

Recumbent or even supine cycling are frequently used alternatives to standard upright cycling (compare Figure 1), due to safety reasons and to avoid movement artifacts in electrocardiograms. By measuring power in watts (W), cycling ergometry provides a theoretical solution for qualifying results among different variants of tests. However, the limited transferability from one position to another (Ray and Cureton, 1991; Bonzheim et al., 1992) must be considered using CPET results as a basis for clinical or performance diagnostics. To provide adequate training recommendations, cycling position must also be taken into account, especially if training and testing postures on the ergometer are different.

It has been shown in several investigations that cycling posture influences circulatory and metabolic outcomes, and therefore also cycling performances (Faria et al., 2005). One of the main reasons for differences between upright and supine cycling seems to be the vertical distance between the active muscles and the heart, which alters the gravitational effect and therefore influences venous return, cardiac output, and perfusion pressure on active muscles (Leyk et al., 1994; Fitzpatrick et al., 1996; Egaña et al., 2010a). Several investigations confirm these differences between supine and upright cycling in terms of higher peak heart rate (HR) and higher maximum performances, time to failure, or maximum power output in upright position. Results differ regarding V_O2__peak_. Moreover, different positions between upright and supine cycling posture, defined by the degree of backward tilt, reveal further outcome deviation compared to upright and supine cycling (Egaña et al., 2010a, 2013).

Furthermore, Leyk et al. (1994) reported a steeper lactate-concentration gradient during incremental exercise tests in a supine position, implying that methods used for training prescriptions [e.g., individual anaerobic threshold by lactate (IAT)] seems also posture-dependent and therefore might require adjustments when testing and training position differs. However, the extent to which blood lactate concentrations during exercise change at different positions remains unclear.

Concerning ventilation, previous studies have demonstrated that supine positions induce reduced ventilation in comparison to upright bicycling. However, to enlighten potential causes for this observation, only little data is known on how different postures influence ventilatory efficiency (Armour et al., 1998; Terkelsen et al., 1999) even though ventilatory efficiency has been proven to be a strong prognostic factor for mortality in patients with cardiac failure (Arena et al., 2007). Both studies compared only upright with supine position. To the best of our knowledge, a recumbent position has not been considered. Furthermore, it has been shown that posture alters Vo_2_ kinetics (Leyk et al., 1994; Koga et al., 1999; Egaña et al., 2013), a factor that potentially reflects metabolic efficiency. However, cycling efficiency have mainly been investigated in constant-load cycling (Hughson et al., 1991; Leyk et al., 1994; Koga et al., 1999; Egaña et al., 2006) rather than during graded exercise tests (DiMenna et al., 2010a), which are usually applied in CPET. Moreover, most studies compared only two postures, while two research groups (Quinn et al., 1995; Egaña et al., 2013) employed additional postures, thus enhancing the informative value of their investigations, but data on cycling efficiency in a recumbent posture is missing at this point.

In clinical practice, especially recumbent cycling positions are commonly used; however results' interpretation is still under debate. Therefore, the aim of this study was to compare metabolic and ventilatory efficiency as well as breathing strategies during cycling in three common working postures [upright, recumbent (tilted backwards by 40°), and supine] to determine how potential differences and results are best assessed and processed for suitable clinical diagnostics and training prescriptions.

We expected that backward tilting would lead to lower peak values and lower submaximal power outputs, according to the literature. New findings will be provided by comparing efficiency in the mentioned three cycling positions, expected to be reduced the further the position is tilted backwards, which may result in increased submaximal Vo_2_ values with lower submaximal power outputs.

## Materials and Methods

### Subjects

Eighteen subjects participated in this study. Recruitment took place via a posting at the University Freiburg. Subjects were recruited through a posting at the university. After reading the study information, all subjects gave their written informed consent. The present study was conducted in accordance with the Declaration of Helsinki and was approved by the Ethics Commission of the University Medical Center Freiburg, Germany and prospectively registered in the German Clinical Trials Register (DRKS00004672).

### Exercise Test

Participants visited our laboratory for three separate testing sessions and completed three incremental cycle exercise tests to exhaustion in upright, recumbent (40°) and supine position (Figure 1). Tests took place in random order on the same day and hour at weekly intervals. Participants were asked to avoid any vigorous exercise 24 h prior to testing sessions and to maintain their usual lifestyle for the duration of the study.

Tests were performed on electronically braked cycle ergometers (upright: Lode Inc., Groningen, Netherlands; recumbent/supine: Ergoline 900, Bitz, Germany), which measure identical power outputs. Both cycle ergometers were calibrated on a regular basis, maintained, and regularly checked for measurement accuracy. Knee angles were measured with a goniometer to maintain constant biomechanical conditions among postures. To avoid large differences in the hip angle, participants were placed in a vertical plane with the upper body upright on the standard upright ergometer (see Figure 1).

Gas exchange and ventilation were continuously recorded by a breath-by-breath gas analysis system (Oxycon Delta, Jaeger, Hochberg, Germany). The volume sensor was calibrated using a 3-L syringe before each test, and gas concentrations were calibrated daily against a reference (16.0% O_2_ and 5.0% CO_2_) and against room surroundings with consideration of humidity, all in accordance with the manufacturer's instructions. Room temperature was constantly regulated to measure 20–21°. After collecting data at rest, exercise tests started at a workload of 20 W and increased by 10 W every minute. Participants chose their preferred pedal cadence between 60 and 70 rpm at the first test, which was maintained and controlled throughout each of the applied settings. Exhaustion was assumed when the subjects were unable to keep within their self-determined pedal cadence (minus 5/rpm) for more than 10 s.

### Data Collection

Heart Rate was measured continuously by electrocardiography (AT 10 plus, Schiller, Baar, Switzerland). At rest and at the end of each exercise stage, 20 μl of capillary blood were taken from the hyperemized earlobe for lactate concentration analysis (Biosen S-Line, EKF-diagnostics, Barleben, Germany). Manually measured systolic and diastolic blood pressure (SBP, DBP) and subjectively perceived exertion (RPE scale 6–20, Borg, 1982) were documented upon termination of the test. Lactate threshold (LT) and IAT were calculated using special software (Ergonizer, Freiburg, Germany). Lactate threshold was defined thereby as the highest exercise intensity before the onset of lactate accumulation (Wasserman et al., 1986), and IAT was described as a net-increase of 1.0 mmol/l above baseline, corresponding to LT (Coyle et al., 1983).

Values of peak Vo_2_ and minute ventilation (Ve) were defined as the mean values during the last 30 s of exercise, whereas maximal tidal volume (V_T_) was taken as the highest mean value for 10-s intervals during exercise testing. Submaximal Vo_2_ and VCo_2_ values were analyzed at LT, IAT, ventilatory anaerobic threshold (AT), respiratory compensation point (RCP), 70 W [Vo_2(70W)_], and at 70% of averaged peak power output [Vo_2(70%)_]. Each subject's AT was identified via the V-slope method (Beaver et al., 1986). RCP was determined at the point of over-proportional increase in the Ve/Vco_2_ plot (Beaver et al., 1986; Meyer et al., 2005) and was visually estimated by two experienced investigators independently. In case of discrepancy, the value used for the analysis was the one with the best agreement. If no agreement was reached, a third independent supervisor made the decision.

Metabolic and ventilatory efficiency were calculated off-line. Ventilatory efficiency is thereby represented by the slopes of linear fittings for Ve/Vco_2_ (Habedank et al., 1998) and Vco_2_/Vo_2_ (Cooper et al., 1992; Honold et al., 2008) from the beginning of the load phase until AT. Aerobic work efficiency is described by the slope of Vo_2_/P until RCP and without onset data to exclude non-linear Vo_2_ behavior (Pokan et al., 1995; Zoladz et al., 1995). Gross efficiency (GE) was determined by applying the following equation (Ettema and Lorås, 2009):

(1) $$\begin{array}{r} GE\left(\%\right)=\frac{\;work\;rate\;\left(W\right)}{energy\;expended}\left(\frac{J}{s}\right)\cdot \;100, \end{array}$$

whereas energy expenditure was calculated as described by Faria et al. (2005) using the formula by Brouwer (1957) that includes a correction against the shifting respiratory exchange ratio (RER) during exercise:

(2) $$\begin{array}{r} \frac{J}{s}=\left[\left(3.869\cdot \overset{∙}{V}O_{2}\right)+\left(1.195\overset{∙}{V}CO_{2}\right)\right]\cdot \left(4.186/60\right), \end{array}$$

GE was calculated at *P*_max_ and at AT and at a workload of 70 W (GE_70w_). We chose 70 W because it was the highest workload at which all subjects remained far below RER of 1.0 in upright position; therefore, this workload represents a mainly aerobic condition with absence of “non-metabolic” CO_2_-production, independent to the individual work capacity.

### Statistical Analysis

Assumptions of normal distribution were verified using the Shapiro-Wilk test. Due to some variables not being normally distributed, differences among postures were analyzed using the non-parametric Friedman test. A *P*-value < 0.05 was considered to be statistically significant. If significant differences were detected, a Wilcoxon signed rank test with Bonferroni correction (α = 0.017) was used for pair-wise comparisons. All data are presented as median, and upper–lower quartile except subject characteristics, which are expressed as mean ± standard deviation (*SD*). Analyses were performed using SPSS statistic version 22.0.

## Results

Eighteen recreationally active (0.5–12.5 h physical activity/week) subjects participated in this study (sex: 10 female, 8 male; age: 47.2 ± 18.4 years; body height: 172.0 ± 8.5 cm; body weight: 74.2 ± 12.8 kg). Subjects with acute infections, acute or chronic pulmonary, or cardiovascular diseases were excluded.

Results of CPET in three different cycling postures are presented in Table 1.

**Table 1:** Results of cardiopulmonary exercise testing in three different cycling postures.

	**Postures**						
	**Upright**		**Recumbent**		**Supine**		***P*-value**
*P*_max_ (Watt)	201	(160–233)	170	(140–211)^a^	165	(138–204)^a^	** <0.001**
HR_max_ (min^−1^)	170	(156–183)^b^	168	(153–190)^b^	158	(144–181)	** <0.001**
Vo_2__peak_ (L**·**min^−1^)	2.64	(2.15–2.89)	2.38	(2.00–2.74)^a^	2.36	(2.03–2.52)^a^	** <0.001**
Vo_2__peak_ (ml**·**kg^−1^**·**min^−1^)	35.0	(27.8–44.6)	31.6	(25.6–42.1)^a^	31.3	(25.2–40.1)^a^	** <0.001**
VCo_2__peak_ (ml**·**kg^−1^**·**min^−1^)	41.6	(34.6–54.2)	38.3	(31.6–50.5)^a^	36.5	(29.2–47.7)^a^	** <0.001**
O_2_pulse_max_ (ml**·**beat^−1^)	15.8	(13.2–17.8)	15.1	(12.1–17.1)	15.4	(12.4–17.3)	0.056
Lact_max_ (mmol^−1^)	8.69	(7.31–9.77)	8.46	(6.81–10.51)	8.81	(7.22–10.00)	0.881
RER_max_	1.18	(1.15–1.21)	1.20	(1.13–1.24)	1.16	(1.11–1.22)	0.311
SBP_max_ (mmHg)	185	(165–205)	205	(170–223)	185	(169–203)	**0.009**
DBP_max_ (mmHg)	90	(80–100)	90	(80–101)	85	(80–96)	0.235
RPE_max_ (score 6–20)	19	(18–19)	19	(17–20)	19	(18–20)	0.249
LT (Watt)	100	(79–114)	77	(68–103)^a^	75	(67–79)^a^	** <0.001**
HR_LT_ (min^−1^)	126	(111–133)	115	(102–125)^a^	112	(104–121) ^a^	** <0.001**
Vo_2(__LT)_ (ml**·**kg^−1^**·**min^−1^)	19.3	(16.6–24.4)	18.2	(16.0–20.4)^a^	17.9	(15.9–19.0)^a^	** <0.001**
VCo_2(__LT)_ (ml**·**kg^−1^**·**min^−1^)	18.4	(15.5–22.3)	16.5	(13.9–20.0)^a^	15.5	(13.7–18.0)^a^	** <0.001**
O_2_pulse_LT_ (ml**·**beat^−1^)	12.3	(10.6–14.0)	12.0	(11.9–13.7)	11.9	(9.8–13.8)	0.080
Lact_LT_ (mmol^−1^)	1.65	(1.17–2.01)	1.46	(1.03–2.02)	1.59	(1.18–1.86)	0.211
IAT (Watt)	136	(113–156)	110	(95–137)^b^	108	(94–119)^b^	** <0.001**
HR_IAT_ (min^−1^)	141	(128–155)	132	(115–145)^a^	122	(116–139)^a^	** <0.001**
Vo_2(IAT)_ (ml**·**kg^−1^**·**min^−1^)	23.5	(20.6–30.9)	22.3	(20.4–26.4)^a^	22.3	(19.4–26.3)^a^	** <0.001**
VCo_2(IAT)_ (ml**·**kg^−1^**·**min^−1^)	24.9	(20.3–30.1)	23.0	(19.2–27.2)^a^	23.0	(18.2–25.4)^a^	**0.001**
O_2_pulse_IAT_ (ml**·**beat^−1^)	13.1	(11.2–15.6)	14.2	(11.1–15.6)	13.1	(11.0–15.4)	0.230
Lact_IAT_ (mmol^−1^)	2.65	(2.19–3.02)	2.47	(2.03–3.03)	2.59	(2.19–2.86)	0.270
AT (Watt)	83	(70–110)	68	(59–90)^b^	65	(50–103)^b^	**0.001**
HR_AT_ (min^−1^)	117	(108–131)	108	(96–126)	109	(99–123)^a^	**0.013**
Vo_2(AT)_ (ml**·**kg^−1^**·**min^−1^)	19.0	(16.4–22.1)	16.5	(15.7–20.0)	17.0	(12.8–21.8)^a^	**0.002**
VCo_2(AT)_ (ml**·**kg^−1^**·**min^−1^)	16.6	(14.4–21.0)	15.5	(12.4–18.0)	15.1	(10.6–18.1)^a^	**0.030**
O_2_pulse_AT_ (ml**·**beat^−1^)	11.5	(10.3–14.7)	11.8	(9.7–14.7)	11.4	(9.2–14.2)	0.486
Lact_AT_ (mmol^−1^)	1.57	(1.02–1.98)	1.51	(0.97–1.87)	1.60	(1.13–1.98)	0.546
RCP (Watt)	170	(148–195)	143	(115–173)^a^	135	(120–160)^a^	**0.006**
HR_RCP_ (min^−1^)	152	(145–171)^b^	150	(133–172)^b^	139	(128–161)	**0.001**
Vo_2(RCP)_ (ml**·**kg^−1^**·**min^−1^)	29.6	(26.2–36.6)	28.0	(22.3–35.4)	26.9	(21.5–32.2)^a^	**0.011**
VCo_2(RCP)_ (ml**·**kg^−1^**·**min^−1^)	34.0	(28.2–41.0)	28.4	(24.2–38.8)	28.8	(21.8–34.5)^a^	**0.008**
O_2_pulse_RCP_ (ml**·**beat ^−1^)	14.9	(11.1–17.0)	14.2	(11.3–17.0)	14.8	(12.1–17.0)	0.127
Lact_RCP_ (mmol^−1^)	5.00	(3.11–5.95)	4.99	(4.12–6.37)	5.08	(3.45–5.57)	0.472
HR_70W_ (min^−1^)	112	(99–126)	112	(95–120)	109	(101–117)	0.741
Vo_2(__70W)_ (ml**·**kg^−1^**·**min^−1^)	16.8	(15.2–18.6)	17.6	(16.2–19.0)	18.1	(15.2–18.7)	0.348
VCo_2(__70W)_ (ml**·**kg^−1^**·**min^−1^)	15.2	(12.8–16.8)	15.4	(13.0–17.7)	15.0	(13.2–17.8)	0.486
O_2_pulse_70W_ (ml**·**beat ^−1^)	11.3	(9.3–12.3)	11.6	(10.3–12.8)	11.9	(9.8–12.9)	0.513
Lact_70W_ (mmol^−1^)	1.47	(1.03–1.87)	1.45	(0.77–1.83)	1.51	(1.08–1.80)	0.946
HR_70%_ (min^−1^)	137	(125–149)	136	(120–157)	135	(121–157)	0.235
Vo_2__(70%)_ (ml**·**kg^−1^**·**min^−1^)	24.0	(21.8–29.2)	25.2	(22.8–30.2)^a^	25.3	(22.9–30.2)^a^	**0.016**
VCo_2__(70%)_ (ml**·**kg^−1^**·**min^−1^)	25.3	(19.5–29.5)	26.6	(22.5–33.2)^a^	26.6	(21.7–34.0)^a^	**0.001**
O_2_pulse_70%_ (ml**·**beat ^−1^)	13.4	(11.0–15.9)	14.3	(11.6–16.9)	14.7	(11.2–17.2)^a^	**0.030**
Lact_70%_ (mmol^−1^)	2.22	(1.94–2.60)	3.46	(2.96–4.08)^a^	3.43	(3.11–4.46)^a^	** <0.001**

*Values presented as median and upper–lower quartile. P-values refer to differences across all three-test conditions. Bold font indicates statistical significance. P_max_, maximum power; HR_max_, maximum heart rate; Vo_2_peak, peak oxygen consumption; VCO_2peak_, peak carbon dioxide production; Lakt_max_, maximum lactate concentration; RER, maximum respiratory exchange ratio; SBP_max_, maximum systolic blood pressure; DBP_max_, maximum diastolic blood pressure; RPE_max_, rate of perceived exertion; LT, lactate threshold; IAT, individual anaerobic threshold; AT, aerobic threshold; RCP, respiratory compensation point; Vo_2_(70 W), oxygen consumption at 70 Watt; Vo_2_(70%), oxygen consumption 70% of averaged peak power output*.

a *Significant difference from upright*.

b *Significant difference from supine*.

Cycling in an upright position resulted in significantly greater *P*_max_ and higher Vo_2peak_ values in comparison with a recumbent and supine position (*P* < 0.001). Moreover, at the submaximal level (70% of *P*_max_) Vo_2_ values were higher when cycling in an upright position (*P* = 0.016). This difference was not detectable under mostly aerobic condition (70 W). Lactate and ventilatory thresholds revealed same responses in terms of increased thresholds in the upright position when individual comparisons between positions were made (LT *P* < 0.001, IAT *P* < 0.001, AT *P* = 0.001, RCP *P* = 0.006). Moreover, in upright position thresholds were detected at higher Vo_2_ and VCo_2._ Lactate concentrations at the peak and submaximal thresholds do not differ between postures.

HR is significantly reduced in supine position (max *P* < 0.001, LT *P* < 0.001, IAT *P* < 0.001, AT *P* = 0.013, RCP *P* = 0.001). Regarding oxygen pulse (O_2_pulse), there are no differences between postures. DBP_max_ did not differ between postures, while a significant difference in SBP_max_ among postures (*P* = 0.009) was seen. However, after pairwise comparison, this difference was no longer significant. RPE values and RER at maximum level did not differ between postures.

### Ventilatory Efficiency

When analyzing ventilatory equivalents for O_2_ (EQO_2_: Vo_2_/Ve) and CO_2_ (EQCO_2_: Vco_2_/Ve) at AT and at the end of the test, no differences appeared among postures. Moreover, estimates of ventilatory efficiency (Ve/Vco_2_ slope) and the Vco_2_/Vo_2_ slope were unaffected by posture (see Table 3). However, when cycling in supine or recumbent position, subjects displayed a trend toward a shallower breathing compared to the upright position: Ve decreased slightly, probably as a consequence of a significant decrease in Vt (Table 2). This change was observed at RCP (Vt_RCP_, *P* = 0.005), at *P*_max_ (Vt_max_, *P* < 0.001), and AT (Vt_AT_, *P* = 0.005).

**Table 2:** Ventilatory strategy in three different cycling postures.

	**Postures**						
	**Upright**		**Recumbent**		**Supine**		***P*-Value**
VE_max_ (L**·**min^−1^)	96.0	(79.1–112.5)	91.8	(76.5–107.4)	88.2	(73.6–101.6)	0.092
VT_max_ (L)	2.33	(2.00–2.91)	2.04	(1.90–2.48)^a^	2.28	(1.82–2.54)^a^	** <0.001**
VFREQ_max_ (min^−1^)	39.7	(32.7–44.7)	43.1	(37.2–47.0)	38.2	(33.2–43.2)	0.223
VE_RCP_ (L·min^−1^)	70.5	(54.8–79.3)	62.8	(48.9–75.8)	62.1	(52.3–63.5)	0.075
VT_RCP_ (L)	2.30	(1.78–2.73)	1.99	(1.77–2.39)^a^	2.00	(1.57–2.56)^a^	**0.005**
VFREQ_RCP_ (min^−1^)	29.0	(26.1–31.4)	30.6	(26.8–34.9)	31.3	(27.0–33.5)	0.472
VE_AT_ (L**·**min^−1^)	35.0	(30.0–44.0)	30.0	(25.8–39.8)^a^	28.5	(25.8–37.0)^a^	**0.025**
VT_AT_ (L)	1.69	(1.44–2.10)	1.54	(1.15–1.79)^a^	1.44	(1.10–2.06)^a^	**0.005**
VFREQ_AT_ (min^−1^)	21.0	(17.5–22.5)	21.0	(18.8–24.5)	22.5	(18.0–25.3)	0.185

*Values are presented as median and upper–lower quartile. P-values refer to differences across all three test conditions. Bold font indicates statistical significance. VE, minute ventilation; RCP, respiratory compensation point; AT, aerobic threshold; VT, tidal volume; VFREQ, ventilation frequency*.

a *Significant difference from upright*.

### Work Efficiency and Gross Efficiency

**Figure 2:**
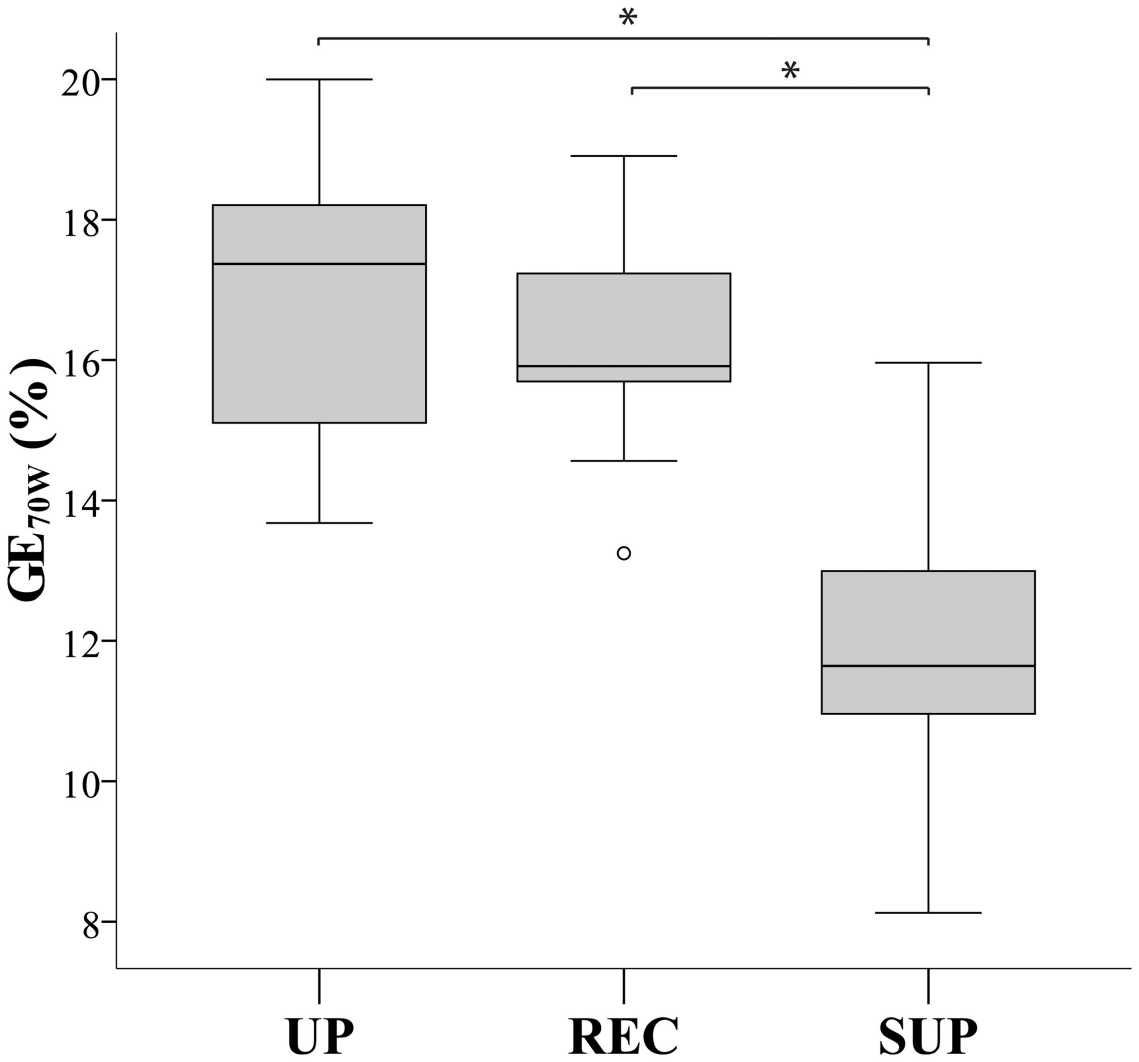
Box plots comparing gross efficiency at 70 Watt (GE_70W_) between cycling postures. UP, upright; REC, recumbent; SUP, supine. *Significant difference (*P* ≤ 0.001).

**Figure 3:**
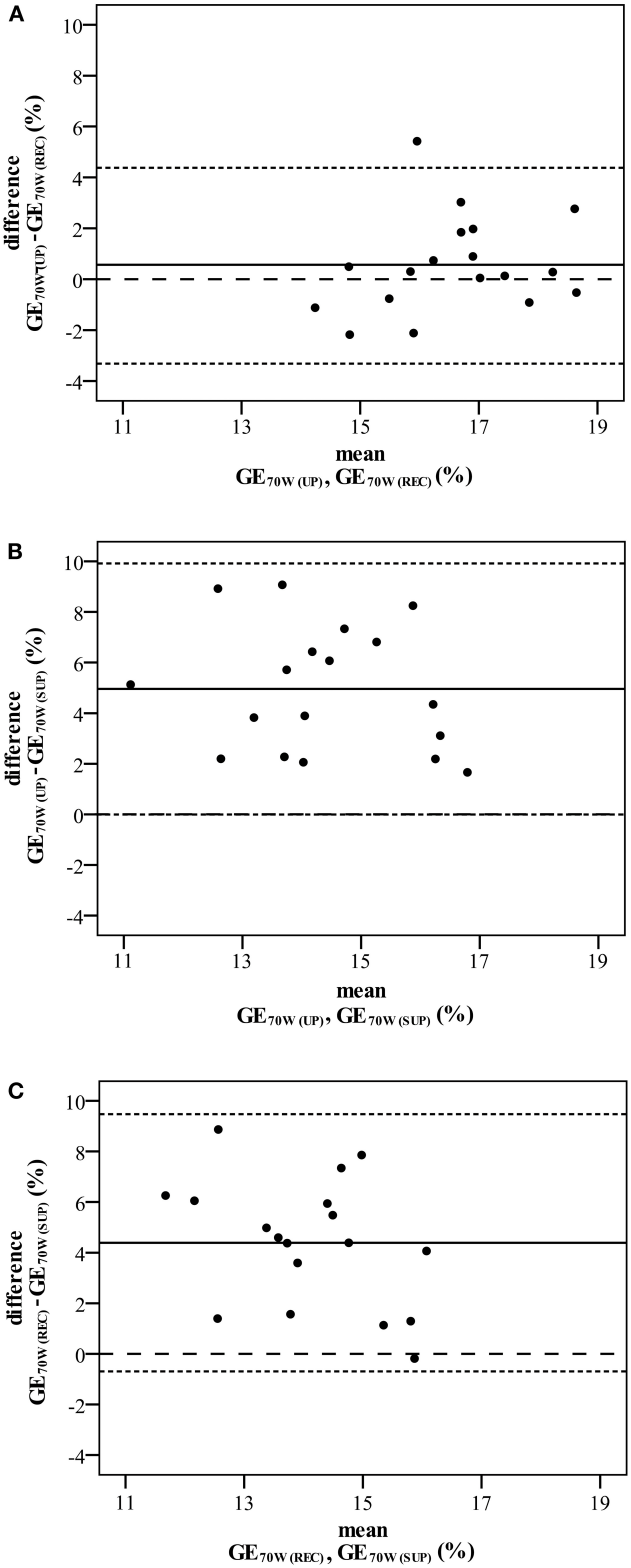
Bland Altman plots comparing gross efficiency at 70 Watt (GE_70W_) during upright and recumbent cycling **(A)**, upright and supine cycling **(B)**, and recumbent and supine cycling **(C)**. The middle solid horizontal lines correspond to the mean difference between postures, the upper and lower dotted horizontal lines represent the 95% limits of agreement given by the mean difference ± 2 *SD*. Dashed lines signify zero differences between postures.

As shown in Table 3, aerobic work efficiency (Vo_2_/*P* slope) was significantly higher during upright than during recumbent and supine cycling (*P* < 0.001). We also noted significant differences in the GE results between postures at different workloads: at *P*_max_ (*P* = 0.002), AT (*P* = 0.034), and at an absolute workload of 70 W (*P* < 0.001). *Post-hoc* analysis indicated that the GEp_max_ in upright cycling was significantly higher than in recumbent cycling, though not in supine cycling. *Post-hoc* individual GE_AT_ comparisons revealed no significant differences between positions, though GE_70w_ in supine posture was significantly decreased in comparison to recumbent and upright postures; *P* < 0.001 (see Figure 2). Bland-Altman plots (Figure 3) illustrate the dimension of the differences across the three different postures.

**Table 3:** Ventilatory and metabolic efficiency in three different cycling positions.

	**Postures**						
	**Upright**		**Recumbent**		**Supine**		***P*-Value**
VE/Vo_2(AT)_	23.6	(20.4–26.5)	23.2	(21.2–25.6)	23.7	(21.9–25.0)	0.801
VE/VCo_2(AT)_	27.2	(22.6–28.6)	27.2	(24.3–29.7)	26.7	(23.7–30.9)	0.066
VE/Vo_2(peak)_	34.2	(32.4–37.0)	34.5	(32.9–39.3)	34.1	(31.7–38.7)	0.389
VE/VCo_2(peak)_	28.9	(27.8–33.6)	30.3	(27.1–33.8)	30.1	(28.7–32.5)	0.211
Slope VE/VCo_2_	25.0	(23.8–26.8)	23.4	(23.2–26.1)	24.9	(23.5–27.1)	0.931
Slope VCo_2_/Vo_2_	0.84	(0.79–0.91)	0.83	(0.67–0.87)	0.79	(0.70–0.88)	0.056
Slope Vo_2_/P (ml·min^−1^·W^−1^)	10.0	(9.5–10.7)	10.8	(10.4–11.9)^a^	10.9	(10.0–12.6)^a^	** <0.001**
GE_Pmax_ (%)	20.6	(20.1–22.1)	19.5	(18.3–21.1)^a^	20.0	(18.7–21.3)	**0.002**
GE_AT_ (%)	18.8	(15.3–20.7)	15.8	(14.7–18.1)	16.9	(14.6–18.7)	**0.034**
GE_70W_ (%)	17.4	(15.1–18.3)^b^	15.9	(15.6–18.3)^b^	11.6	(10.9–13.3)	** <0.001**

*Values are presented as median and upper–lower quartile. P-values refer to differences across all three test conditions. Bold font indicates statistical significance. VE, minute ventilation, AT, aerobic threshold; P, power; GE_Pmax_, gross efficiency at peak power output; GE_AT_, gross efficiency at aerobic threshold; GE_70W_, gross efficiency at 70 W*.

a *Significant difference from upright*.

b *Significant difference from supine*.

## Discussion

The main and novel findings of this study are two-fold. Firstly, cycling efficiency decreased by tilting the body in a recumbent or supine position in terms of decreased aerobic work efficiency; GE under a predominantly aerobic condition was significantly reduced in supine position. Secondly, ventilatory efficiency is surprisingly not affected by posture, though breathing was shallower in recumbent and supine positions with significantly decreased V_T_.

### Efficiency and Breathing Strategy

Vo_2_/P slope values were significantly higher in supine and recumbent positions than in the upright position. This appears to concur with previous findings, which showed upward curvilinearity above AT in supine cycling (Koga et al., 1999; DiMenna et al., 2010a). This non-proportional increase in the oxygen cost of work, that could now also be shown in a recumbent position, can be attributed to the additional work of assisting muscles at higher workloads, e.g., trunk and breathing muscles (Jones et al., 2011). Additionally, in the recumbent or supine position, the support provided by the subject's body weight is less or completely absent. The force required to move the pedal must be provided here entirely by dynamic contraction and less by static holding, which can explain a large part of this difference. The augmented contribution of type II muscle fibers in exercises above AT (Barstow et al., 2000; DiMenna et al., 2010a; Jones et al., 2011) and therefore, the higher O_2_ cost of ATP production, may also be responsible for the steeper slope and, in particular, showed an impact on unfamiliar and inefficient cycling positions. Additionally, an altered muscle perfusion due to supine (Koga et al., 1999; DiMenna et al., 2010b) or recumbent position is assumed. The slope was not substantially affected by potential upper body work due to gripping the handlebars in an upright position. Probably the influence of body weight support already described above can play a counterbalancing role.

Interestingly GE_70W_ was significantly reduced in supine position in the present study, revealing that body tilt affects efficiency significantly. Even though a low metabolic load at 70 W for all tested subjects, our results regarding GE_70W_ may already provide an indication of the muscular fatigue at an early stage of the test. Some other workgroups presented evidence that the rate of fatigue during exercise increased when the body is tilted in a supine or recumbent position (Fitzpatrick et al., 1996; Egaña and Green, 2007). More specifically, Egaña et al. (2010b) confirmed these results during high-intensity constant-load cycling in supine vs. upright posture, and this higher rate of fatigue was accompanied by increased muscle activation (Egaña et al., 2010b). In fact, this reflects the higher energy cost during supine cycling and may explain the reduced efficiency in recumbent and supine postures in this study. Although the net mechanical gravitational contribution to pedal power is minimal, projection of the center of gravity, and therefore a higher counterfort due to body weight in upright posture as well as a different muscular activation pattern (Brown et al., 1996), might have influenced GE. For deeper insight into the mechanisms behind the different responses, the use of other non-invasive techniques (e.g., near-infrared spectroscopy, electromyography) could be considered in future investigations.

In line with several other studies (Bonzheim et al., 1992; Quinn et al., 1995; Egaña et al., 2013) no differences could be observed between postures regarding Ve at higher workloads, whereas Ve at AT was significant higher in upright position. Interestingly breathing became shallower from one posture to another. Recumbent and/or supine cycling led to significant lower Vt, and consequently breathing frequency must be adapted to maintain ventilation (that should have induced more wasted ventilation to the deadspace). It was assumed that this was an indication of possible mechanical constraints caused by body position (Romei et al., 2010) reinforced by incomplete leaning against the back rest. Nonetheless, the ventilatory efficiency results (Ve/Vco_2_ slope) remained unchanged among postures, indicating that ventilation and therefore metabolism/oxygen delivery is not influenced by position. This seems also clinically relevant, since exercise tests in recumbent and even supine positions have no significant influence on this clinically measured value and therefore seems not to result in a falsely negative diagnosis.

### Impact of Posture on Peak Values and Metabolic Thresholds

Our HR findings replicate those from previous studies, as we also noted higher values in upright and recumbent than in supine posture. O_2_pulse at maximum and at the thresholds does not change, an indication that the stroke volume does not change with posture. Only at 70% of the averaged peak power is a significant change evident: a lower O_2_pulse in supine position, indicating a higher metabolic load, which is also reflected by the lactate, Vo_2_ and Vco_2_ values. Statistical differences among postures in SBP_max_ lost their validity after pairwise comparisons. This in fact has an important role to play in clinical practice: when maximum blood pressures in different postures are similar despite higher *P*_max_ in upright cycling, upright cycling should be preferred in certain situations, in particular when physical stress should be avoided.

As mentioned before and according to other studies, *P*_max_ and Vo_2peak_ were higher in upright than in recumbent and supine position (Proctor et al., 1996; Egaña et al., 2006, 2010a, 2013; DiMenna et al., 2010a; Kato et al., 2011). However, some investigators reported no difference in Vo_2peak_ (Bonzheim et al., 1992; Quinn et al., 1995) or even opposite results regarding *P*_max_ (Bonzheim et al., 1992). Those investigations were carried out in patients suffering from cardiac diseases, which could have yielded deviating results as their patients probably failed to achieve complete exhaustion. The present results reveal decreased values in recumbent and supine positions, which may indicate that muscular fatigue, and therefore exhaustion, appeared earlier at a lower workload and thus peak values could not attain the same level as in the upright position. Furthermore, the abovementioned additional upper body work due to gripping the handlebars in upright position could also have caused higher Vo_2peak_ values (Stenberg et al., 1967). Thus, when classifying individual test results, influences of cycling position must be kept in mind.

Circulatory responses at threshold references (respiratory and lactate) were significantly higher in the upright position in our study—a finding that appears highly relevant in terms of its implications for clinical practice. From the perspective of therapeutic exercise, it seems important that the training load calculated by values measured in a specific posture needs to be adjusted when exercising in another posture to ensure that the training stimulus is adequate. Moreover, there are indications that posture may affect cardiorespiratory adaptations to cycle training (Ray and Cureton, 1991).

## Conclusions

The present study demonstrates for the first time the effect of posture on cycling efficiency not only in supine or upright cycling position, but also in a clinically relevant recumbent position during graded exercise testing.

The results show higher power outputs and increased cycle efficiency in upright cycling in comparison to recumbent and supine posture, though ventilatory efficiency was less affected by posture. This therefore indicates that CPET can be performed in supine or recumbent position without triggering falsely negative results regarding prognostic ventilatory values like Ve/Vco_2_ slope. However, when considering submaximal and peak performances as prognostic values, it is essential to consider the differences due to posture. Our result also reinforces the importance of adjusting test results—depending on testing positions—when prescribing training programs to ensure an adequate training stimulus. Thus it is essential to mention the test position when reporting CPET results.

## Data Availability Statement

The original contributions generated for the study are included in the article/supplementary material, further inquiries can be directed to the corresponding author/s.

## Ethics Statement

The studies involving human participants were reviewed and approved by Ethics Commission of the University Medical Center Freiburg, Germany. The patients/participants provided their written informed consent to participate in this study.

## Author Contributions

AW, SW, and KR contributed to the conception and design of the study. AW and SW were responsible for data collection and interpretation. AW drafted the manuscript. KR supervised the measurement and provided assistance for the data analysis and interpretation as well as drafting of the manuscript. AG revised the manuscript. All authors contributed to the article and approved the submitted version.

## Conflict of Interest

The authors declare that the research was conducted in the absence of any commercial or financial relationships that could be construed as a potential conflict of interest.
